# Aerial Laser Scanning Data as a Source of Terrain Modeling in a Fluvial Environment: Biasing Factors of Terrain Height Accuracy

**DOI:** 10.3390/s20072063

**Published:** 2020-04-07

**Authors:** Zsuzsanna Szabó, Csaba Albert Tóth, Imre Holb, Szilárd Szabó

**Affiliations:** 1 Department of Physical Geography and Geoinformatics, University of Debrecen, Doctoral School of Earth Sciences, Egyetem tér 1, 4032 Debrecen, Hungary; 2 Department of Physical Geography and Geoinformatics, University of Debrecen, Egyetem tér 1, 4032 Debrecen, Hungary; toth.csaba@science.unideb.hu (C.A.T.); szabo.szilard@science.unideb.hu (S.S.); 3 Institute of Horticulture, University of Debrecen, Böszörményi út 138, 4023 Debrecen, Hungary; holb@agr.unideb.hu

**Keywords:** floodplain, noise filtering, interpolation, cloth simulation filter (CSF)

## Abstract

Airborne light detection and ranging (LiDAR) scanning is a commonly used technology for representing the topographic terrain. As LiDAR point clouds include all surface features present in the terrain, one of the key elements for generating a digital terrain model (DTM) is the separation of the ground points. In this study, we intended to reveal the efficiency of different denoising approaches and an easy-to-use ground point classification technique in a floodplain with fluvial forms. We analyzed a point cloud from the perspective of the efficiency of noise reduction, parametrizing a ground point classifier (cloth simulation filter, CSF), interpolation methods and resolutions. Noise filtering resulted a wide range of point numbers in the models, and the number of points had moderate correlation with the mean accuracies (r = −0.65, *p* < 0.05), indicating that greater numbers of points had larger errors. The smallest differences belonged to the neighborhood-based noise filtering and the larger cloth size (5) and the smaller threshold value (0.2). The most accurate model was generated with the natural neighbor interpolation with the cloth size of 5 and the threshold of 0.2. These results can serve as a guide for researchers using point clouds when considering the steps of data preparation, classification, or interpolation in a flat terrain.

## 1. Introduction

Digital terrain models (DTMs) are effective and important tools of environmental investigations, engineering, and planning [1,2,3]. DTMs are often used for the management of natural risks, e.g., assessments of inundation exposure or volcanic active areas, especially if these areas are populated and involve infrastructure [4,5,6] There are several ways to produce these models, such as interpolating surfaces from surveyed field data or vectorized contours of maps and using the principles of stereo photogrammetry (airborne and satellite), SfM (structure from motion) technique; the most dynamically developing technique is the application of airborne LiDAR/ALS (LiDAR—light detection and ranging; ALS—airborne laser scanning), which provides a three-dimensional point cloud stored in binary LAS (LiDAR archive standard) format [7,8,9,10,11,12,13,14,15]. The ALS technique has the potential to collect multilayer data including the ground points if laser beams can reach the bare earth: emitted beams have echoes (reflections or discrete returns) from the top of the objects as the “first echo” and from the ground as the “last echo”, and of course there are internal echoes as well [16,17]. However, the last echoes do not always reach the ground; consequently, generated terrain models can have a bias [18]. In spite of the difficulties, there could be several ways to filter out the noise and the ground points of a three-dimensional point cloud before DTM generation; therefore, the final results can be improved and can be used for most purposes.

Noise is considered to consist of outliers which have different characteristics than the neighboring points, i.e., supposing a locally planar area (i.e., kernel window) defined by the average distance from a center or considering its *k* neighbors, when outlying points fall outside it. Noise filtering can be based on principal component analysis [19], neighborhood distance [20,21], or distance from surface [22].

Ground point filtering is also a crucial point of data preparation [13]. Several types of filtering algorithms have been developed for the extraction of ground points automatically. Some algorithms also apply the kernel approach and compare points to their neighbors (binning) assuming that the minimum values of the kernel window represent the ground [23] or that slopes cannot exceed a given angle value within the kernel [24]. Besides the fixed kernel windows, other robust methods exist, including the iterative multiscale spline, which was developed directly for densely forested areas [25]. Furthermore, [26] used a progressive morphology, [27] used a segmentation-based robust interpolation, and [28] used a combination of a multilevel adaptive filter (MAF) with morphological reconstruction and a thin plate spline (TPS) interpolation algorithm for the classification procedure to extract more precisely the bare earth. In addition, nowadays, there is an increasing number of easy-to-use algorithms which are implemented in freely available software to help for the users with the filtering process. For example, [29] has promoted a new method, the cloth simulation filter (CSF), implemented in many open-source software, e.g., Python [30] and CloudCompare [31], which extracts ground points by simulating a physical process in which a virtual cloth covers the inverted point cloud. Its advantages are that it can be used for various landscapes and the parameters are easy to set.

Floodplains represent a complex environment as they are important from the perspective of flood management, agricultural production, and nature conservation [32,33,34]. An accurate DTM can be exploited to delineate trajectories of moving water during high and retreating floods, to find appropriate places for ploughing or animal grazing, to identify environmental conditions which provide good habitats for valuable species, or even to help with the detection of heavy metal hotspots [2,4,35,36,37,38]. However, the most important locations of floodplains are usually impervious places with dense vegetation combined with permanent or periodical water cover (swales, oxbow lakes, peats, or marshes) [39]. Accordingly, field surveys cannot be effective; furthermore, photogrammetry also has problems due to the vegetation cover and its ability to produce only a digital surface model (DSM) [40,41]. The ALS technique is also limited, as beams do not always reach the ground due to dense vegetation, and water cover on the landforms will absorb the emitted light. Accordingly, point clouds always have a bias in a complex environment where the vegetation and/or the topography impede the penetration of laser beams to the ground. However, despite these issues, this is the most effective method of data collection in these areas [42,43], and pre- and postprocessing and different classification procedures can mitigate the errors [44].

The floodplain of a typical meandering river is usually characterized by concave or convex shapes, i.e., deeper or higher terrains with specific characteristics known as swales and point bar series [45,46]. Swales are shallow concave forms often covered with herbaceous (e.g., reed) or aquatic vegetation (e.g., common duckweed) [47]. Point bars are the antonyms of swales; they have a convex shape and are higher. In the floodplain of a medium-sized river, the height differences are relatively small (1–5 m), but a swale–point bar series usually has a relative difference of 0.5–1 m. This small relief highlights the main problem of the surveys: a small error has relevant consequences in the final terrain model and in all the extracted secondary information. Therefore, digital terrain modeling is a great challenge in this flat environment as small errors are conspicuous and fluvial forms become distorted. Common methods which fit for hilly and mountainous areas can fail, and postprocessing (e.g., filtering of the DTM itself, sink fill) can be misleading, diminishing real phenomena (even the forms themselves).

In this study, we aimed to reveal the efficiency of different denoising approaches and the fine-tuning of CSF as a ground point classification technique. We intended to conduct LAS data processing in open-source environment and to use the most widespread algorithms. Although there are several research studies presenting details and suggestions concerning the best practice to generate DTMs [13,48,49], there has been no comprehensive analysis of how the multiple factors of noise filtering, ground point classification, interpolation techniques, DTM resolution, and fluvial geomorphology together influence the accuracy of the models. Finding the DTM the most precisely reflects the terrain characteristics is crucial in such a flat environment where even a centimeter-scale error can change the topography and therefore change the determination of the waterflow direction (flood risk management), sediment accumulation (floodplain land use management) or the identification of fluvial forms. In this work, our aim was to evaluate and quantify the differences of point cloud classification algorithms and to compare the resulting DTMs. As ALS provides a high density of information about the terrain, deterministic interpolation methods were used and compared here. Our hypotheses were the following: (1) ground points have a significant effect on the generated DTMs, and filtering methods decrease the errors to a relevant degree; (2) ground point identification is highly dependent on the cloth size and the threshold parameters of CSF; (3) interpolation techniques and the grid size can enhance or smooth the errors of the DTMs; (4) different morphological forms, in our case swales and point bars, have a significant effect on ground point density.

## 2. Study Area and Topographic Characterization

**Figure 1:**
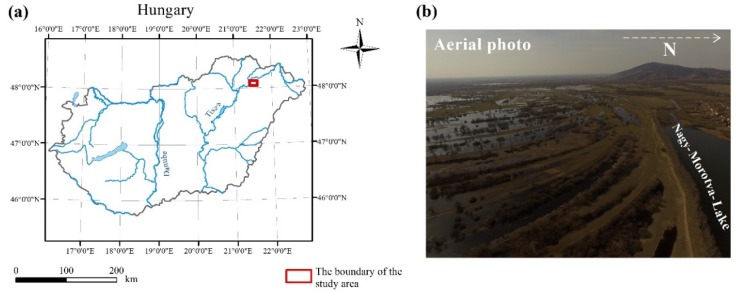
(**a**) The location of the study area; (**b**) a photo from the studying area showing point bar and swale series, captured by drone by Csaba Albert Tóth, 2017.

**Figure 2:**
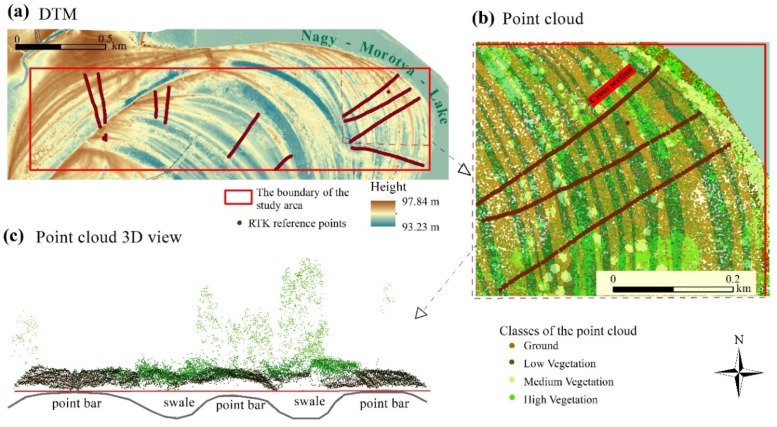
The characteristics of the study area. (**a**) It is characterized by a well-developed point bar and swale series; (**b**) the widths of bars and swales are various; (**c**) the point cloud of the study (exaggeration: 4) reflects back that swales usually have denser vegetation than point bars.

The study area is situated near to the town of Rakamaz, in NE Hungary (the coordinates of its corners are as follows: upper left 48°7’6.8226” N 21°26’37.1286” E, upper right 48°7’6.3942” N 21°28’24.8376” E, lower right 48°6’48.0528” N 21°28’24.672” E, lower left 48°6’48.4806” N 21°26’ 36.9702” E), in the floodplain of the Tisza River (Figure 1a). It covers approximately 1 km^2^ and is characterized by point bar and swale series (Figure 1b, Figure 2a), which has remained as a consequence of the continuous lateral movement of the former Tisza River bed. It was selected due to its diverse environment. The widths of these landforms are various, mostly ranging between 10 and 30 m, but there are some narrower ones (3–5 m), and some are really well spread, with a width of more than one hundred meters. Differences in terrain height in most of the cases are less than one meter (0.3–1 m) between the point bars and swales situated next to each other. The different morphology of the two landforms—point bars are positive, swales are negative forms—cause essential discrepancies: e.g., the ground water level is closer to the surface in the swales due to their concave shapes, and also precipitation and snowmelt run off from the concave form and gather here (Figure 1b). Besides, they have differences in their granulometric composition, with swales having finer sediments that also slow down the infiltration of the water [46]. All these features provide a higher percentage of moisture in swales, which support denser vegetation (that in some cases becomes impervious) and afford good conditions for aquatic vegetation (reed, sedge, etc.). In contrast, the vegetation density of point bars is relatively sparse compared to swales, except when they lie in a relatively lower part of the floodplain, because in this case their surface can be also covered by dense reeds. In Figure 2b,c, we highlight the pattern of the vegetation as it is shown in a portion of the 3D view of a point bar and swale series. The density of the vegetation points is higher and that of the ground points is lower in the case of the swales. In our previous work [50] we also quantified this fact.

The permanent and temporary water surface, the sedge-marsh and reeds, and the pastures and grazing lands make up the landscape mosaic of the study area and provide valuable habitats. All the floodplain here belongs to the Natura 2000 network and Ramsar sites.

## 3. Materials and Methods

### 3.1. Aerial LiDAR Dataset

The study area was surveyed by a RIEGL LMS-Q680i ALS LiDAR fixed on a Cessna C-206 Skywagon aircraft on 20 August, 2012 (Table 1) in the framework of a project to prevent and manage natural disasters [51].

**Figure 3:**
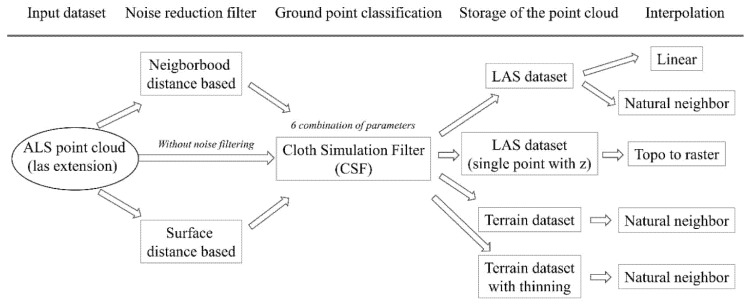
The workflow of the analysis (full factorial analysis in all possible combinations, altogether there were 180 models).

The georeferenced point cloud of the study area, which was our basic dataset for the input of the analysis (Figure 3), was produced by Envirosense Ltd. and provided by the Trans Tisza Water Directorate.

### 3.2. Data Preparation

We applied two types of noise reduction filters for all the points of the point cloud data (Figure 3). Both filters apply a fixed kernel window, which assigns a certain number of points. Points delineate a small local plane area and the algorithm excludes the points exceeding a threshold value based on the distance between the delimited points (Section 3.2.1) or the distance from the surface (Section 3.2.2). As these filters use mean and standard deviation, they are called statistical outlier removal (SOR) filters. The analyses were carried out in CloudCompare 2.10.2 software [31].

#### 3.2.1. Neighborhood Distance-Based Filter

The neighborhood distance filter is a kernel-based, neighborhood-related approach where the user-defined *k* nearest neighbors are investigated by each point of the dataset to see whether there are points exceeding the average distance plus standard deviation (Equation (1)).

(1) $$maximum\;distance={\underline{d}}_{k}+n\sigma \;[-]$$

where ${\underline{d}}_{k}$ denotes the average distance of the *k* neighbors around a given point (center); σ is the standard deviation of the distances from the center; and *n* is the user-defined parameter, usually with a value of 1–3.

We followed the recommendations and performed the noise filtering with 8 neighboring points (*k*) and with 2*σ*.

#### 3.2.2. Surface Distance-Based Filter

This filter also uses a kernel window based on the user-defined number of neighboring points on a search radius, but outliers are selected calculating the distance from the local surface within the kernel (Equation (2)). Noise can be identified similarly to Equation (1), but the distance from the surface, or absolute maximum, can also be defined. We can exclude isolated points, where the number of neighbors is less than 3.

(2) $$maximum\;distance={\underline{sd}}_{k}+n\sigma \;[-]$$

where ${\underline{sd}}_{k}$ denotes the average distance from a local planar surface defined by k neighboring points around a given point (center); σ is the standard deviation of the distances from the planar surface; and *n* is the user-defined parameter. If we choose a relative error, it is usually with a value of 1–3 (to exclude the statistical outliers).

We applied the relative error option with 8 neighbors, 2σ, and excluding island points.

#### 3.2.3. Ground Point Classification

We applied the CSF developed by [29] to identify the ground points. Cloth refers to the grid size covering the study area, and higher values result in coarse DTMs. The procedure works “by analyzing the interactions between the cloth nodes and the corresponding LiDAR points, the locations of the cloth nodes can be determined to generate an approximation of the ground surface” [29]. There are three types of terrain that can be considered in terms of their surface rigidness and defined by users: mountain areas with steep slopes, hilly areas understood as complex landscapes with trees and houses, and flat areas with high houses.

CSF parameters are dependent on the point cloud density (cloth size, i.e., resolution) and the complexity of the terrain (threshold, i.e., the distance between points and the simulated terrain), and although suggestions exist, there is no definite rule for setting these parameters. Rather, a range can be used to find the ideal ones. We conducted the classification with the flat terrain option (as the relief was very low in the study area) and with cloth sizes of 2 and 5 (the larger the cloth, the coarser the DTM), according to [29]. A value of 2 was suggested based on our point density, but we also intended to test the effect of a larger value (i.e., 5) We used classification thresholds of 0.2, 0.5, and 1, with 500 iterations in each case. The CSF was performed in CloudCompare 2.10.2 [31].

### 3.3. DTM Generation

We loaded the LAS files of the ground points into: (1) the LAS dataset, which is most commonly used for storing LAS files; (2) the terrain dataset (TD), which is a multiresolution, TIN-based (triangular irregular network) surface assembled from the ground points; and (3) a single point with z information. The different storage methods offered different opportunities to produce digital terrain models. In the case of the terrain dataset, two building options were used: the first was without thinning (z-tolerance), abbreviated as TD; the second was with the Z minimum point selection method and the moderate secondary thinning method with a threshold of 1 [52], abbreviated as TH. We expected that the secondary thinning could improve the final models’ accuracy, as it could thin points which were ‘far’ from the minimum.

For the DTM production three interpolation methods were used: (1) natural neighbor interpolation with the minimum cell assignment type (NA); (2) linear interpolation with the nearest neighbor cell assignment type (LI); and (3) topo to raster (TT). Each of them was generated with two cell sizes, 1 m and 2 m, as these seemed to be the most appropriate pixel sizes due to the LiDAR point density and the width of the landforms. The natural neighbor is a weighted-average method using Thiessen polygons for the analysis of proximity to determine a cell value [53]. The linear interpolation assigns the z values from the plane determined by the surface triangle that contains the x,y coordinates of a given point [52]. The topo to raster creates a hydrologically correct raster from the points using the ANUDEM algorithm (elevation gridding method) developed by [54,55]. Altogether, 180 DTMs were generated. All DTMs were produced in Esri ArcGIS 10.3 software with 3D Analyst and Conversion Tools [52].

### 3.4. Validation and Statistics Analyses

A field survey was carried out by a Stonex S9 RTK (real-time kinematic) GPS (global positioning system) using the “stop-and-go” method with a real-time differential correction of the GNSS (global navigation satellite systems) permanent station system of Geotrade Ltd., Hungary. The accuracy was ±0.01 m both vertically and horizontally. A total of 604 reference points were taken crossing 8–15 swale–point bar series (Figure 2a). The dataset was used to validate the models. We defined the term of accuracy as the difference between the reference measurements and the modeled data.

We extracted the values of all models where ground control measurements were available (604 RTK points) and subtracted them from the measured values of the RTK points. Finally, considering all factorial combinations (with 4 factors), 107,937 pieces of data were available in the analysis (Supplementary material). We analyzed the dataset in terms of the efficiency of noise reduction, the different settings of CSF, the interpolation method, and the resolution. Finally, we also examined the effects of the different combinations of data preparation and interpolation techniques on the accuracy of the representation of fluvial forms, i.e., how the point bars and swales can be modeled and which approach results in the most accurate model.

Spearman correlation (r) was used to analyze the correlation between the number of points and the model accuracies gained; we reported correlation at p (significance) < 0.05. This type of correlation does not suppose a normal distribution and is not influenced by recurring similar data [56].

We applied the Welch test for one-way comparisons with the Tukey HSD (honestly significant difference) post hoc test. The Tukey test is not sensitive to normal distribution [57]. A robust two-way factorial ANOVA (analysis of variance) using 20% trimmed means and bootstrapping (999 replications) was used to reveal the interactions between the factor variables. As a result of the trimming, the analysis was not sensitive to outlier data. Based on the bootstrap approach—i.e., generating several replications, in our case 999—with random sampling from the original dataset, statistical parameters can be calculated related to the prediction error, variance of mean, etc. Accordingly, this robust approach does not require normal distribution [58].

Swales and point bars were analyzed with the Wilcoxon test with Monte Carlo analysis (with 99,999 repetitions) [59]. The H_0_ was that there was no difference between the number of points per square meter of the fluvial forms.

Statistical analyses were conducted in R 3.53 statistical software [60] with the coin [61], the onewaytest [62] and the WR2 [63] packages.

List of statistical abbreviations used in the results paragraph:

- df: degree of freedom
- F: F-statistic
- p: significance
- pmc: Monte Carlo simulation based p-value (significance)
- Q: Q-statistic for 2-way ANOVA (analysis of variance)
- r: Spearman correlation
- W: Wilcoxon test statistic
- z: z-score

## 4. Results

### 4.1. Number of Points and Accuracy

Noise reduction resulted in a smaller number of points, reducing the input data of the ground point classification from 10.1 million to 8.7 million (Table 2). The original dataset—without noise filtering—contained several points which caused false terrains in the final models.

The different parameters of the CSF provided ground points on a large scale from 8.3 million to 3.7 million. Thus, the difference was large in the models, and the number of points had a moderate correlation with the mean accuracies (r = −0.65, *p* < 0.05), indicating that smaller points provided better agreement with the field measurements. The smallest differences belonged to the neighborhood-based noise filtering, the larger cloth size (5), and the smaller threshold value (0.2). Generally, the differences were low, between 0.09–0.16 m, but the standard deviations were high (0.13–0.18 m), indicating high relative standard deviation (even more than 100%).

### 4.2. Effect of Noise Reduction and the CSF Parameters

**Figure 4:**
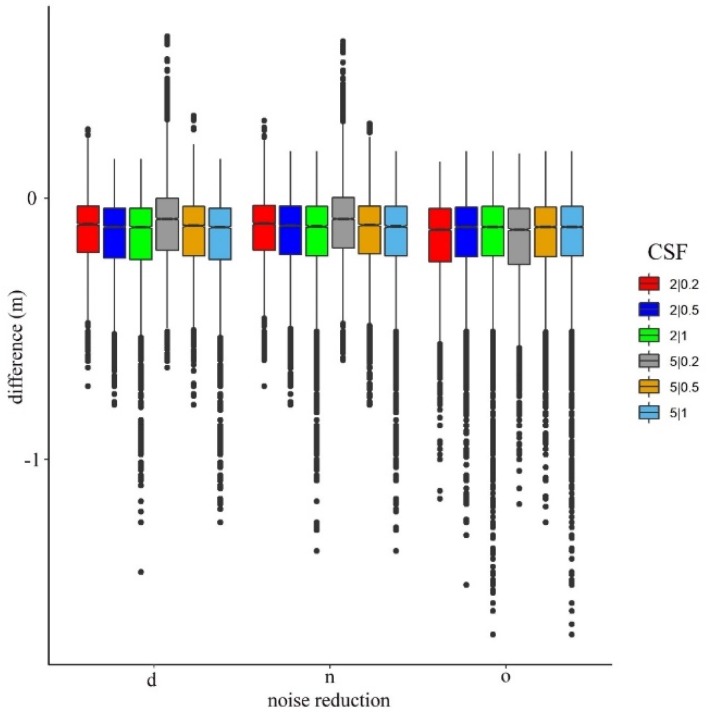
Difference between ground reference and the modeled values by noise filtering methods (d: surface distance-based, n: neighborhood distance-based filtering, o: original point cloud) and cloth simulation filter (CSF) settings (cloth size | threshold).

Considering the noise reduction itself, according to the Welch test, all models had significant differences (F = 194.1; df = 7.18 × 10^−4^; *p* < 0.001). In the following step, we analyzed the effects of CSF parameters, the cloth sizes, and thresholds (Figure 4). The smallest differences were obtained using the point clouds with noise reduction and the cloth size of 5, and a threshold of 0.2 occurred in the models (the difference was −0.08 m in relation to the reference). In the case of the original point cloud, these parameters provided the worst model, having the poorest accuracy (−0.12 m).

**Figure 5:**
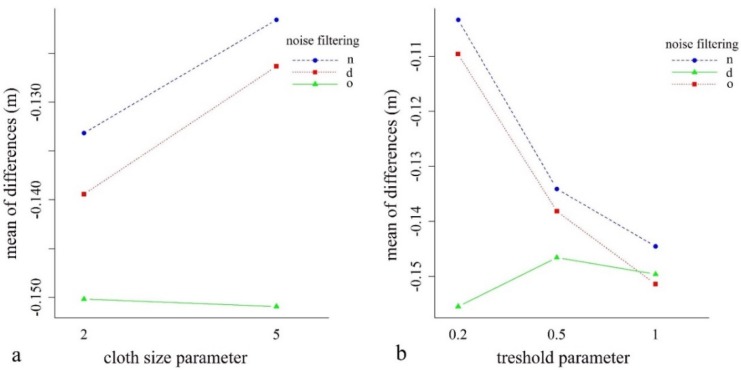
Mean differences (m) between the cloth size (**a**) and the threshold (**b**) parameters according to the noise filtering (d: surface distance-based, n: neighborhood distance-based filtering, o: original point cloud).

The two-way factorial ANOVA revealed the relevance of the noise filtering and the CSF parameters (Figure 5). The results confirmed the observations from Figure 4, i.e., that both the noise filtering and the specifications of the CSF parameters had a significant effect on the modeled values. There were significant main effects for noise reduction (Q = 147.8; *p* < 0.001), for cloth size parameters (Q = 19.65; *p* < 0.001), and for their interaction (Q = 17.82; *p* < 0.001). We observed a similar case with the model of threshold values: the main effects were significant (for noise Q = 149.1, *p* < 0.001; for threshold Q = 87.67, *p* < 0.001), and the interaction of noise reduction and threshold was also significant (Q = 231.3, *p* < 0.001).

### 4.3. Consequences of the Interpolation Algorithms

**Figure 6:**
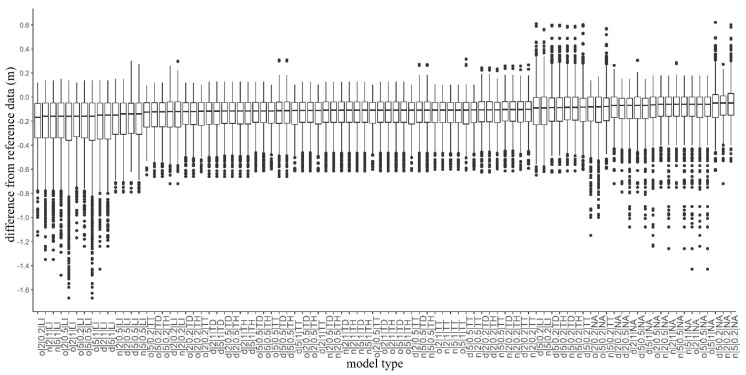
Boxplots of model accuracies ordered by medians for 1 m resolution (o: original database, d: distance-based noise filter, n: neighborhood-based noise filter; first number: cloth size parameter; second number: threshold parameter; LI: linear interpolation, NA: natural neighbor interpolation, TD: terrain dataset with natural neighbor interpolation; TH: terrain dataset with thinning and natural neighbor interpolation; TT: topo to raster interpolation).

**Figure 7:**
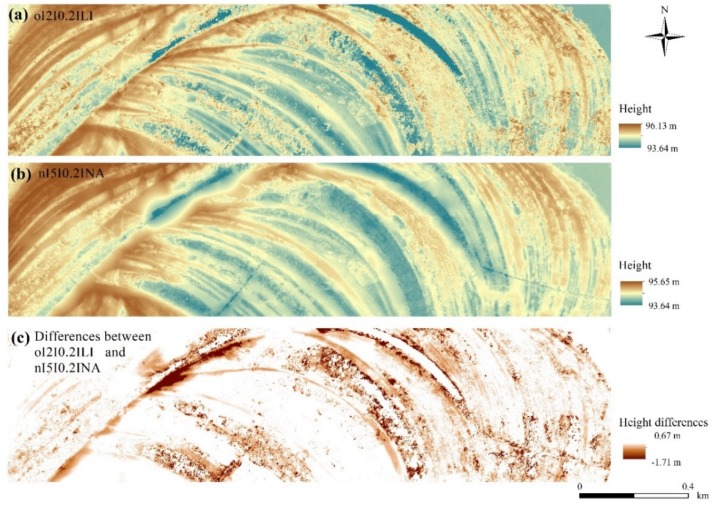
The best and the worst models according to the range of medians and the differences between them: (**a**) o|2|0.2|LI: o: original database; first number: cloth size parameter (2); second number: threshold parameter (0.2); LI: linear interpolation; (**b**) n|5|0.2|NA: n: neighborhood-based noise filter; first number: cloth size parameter (5); second number: threshold parameter (0.2); NA: natural neighbor interpolation. (**c**) The difference between o|2|0.2|LI and n|5|0.2|NA terrain models.

**Figure 8:**
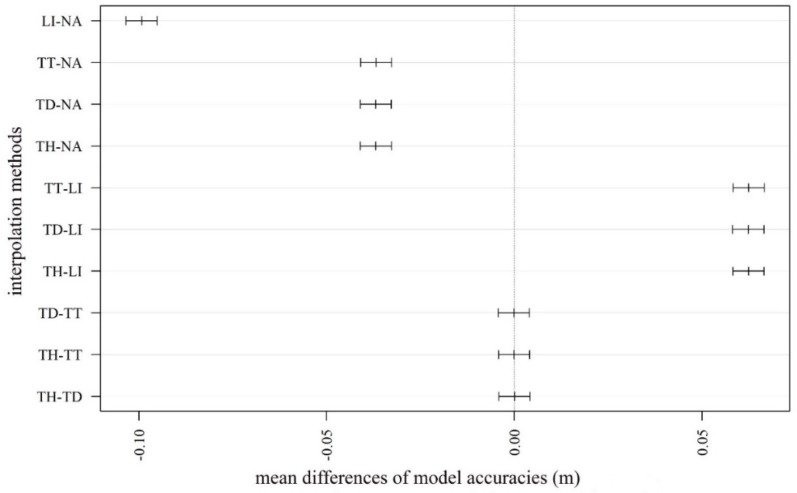
Mean differences by interpolation types (LI: linear interpolation; NA: natural neighbor interpolation; TD: terrain dataset with natural neighbor interpolation; TH: terrain dataset with thinning and natural neighbor interpolation; TT: topo to raster interpolation; error bars: 95% confidence intervals; insignificant differences: where error bars intersected the 0 value, this is shown by the vertical dashed line).

Considering the medians of the accuracies, values ranged between −0.16 and −0.05 m (Figure 6). However, the quartiles and the outliers varied on a larger scale (minimum: −1.67 m; maximum: 0.619 m) indicating that modeled values were influenced by several factors. Subtracting the worst from the best model, the largest difference in modeled height was −1.71 m. The visual interpretation highlighted that the model without filtering, using inappropriate CSF parameters and interpolation techniques, resulted in a DTM with a high number of pixels with noise. The noise is mainly concentrated in the area with higher density of vegetation (we will consider this issue more deeply in Section 4.5) (Figure 7). Generally, interpolations resulted in differences between the model pairs, except in the cases of TT, TD, and the terrain dataset with thinning TH methods (F = 745.8; df = 5.35 × 10^−5^; *p* < 0.001; Figure 8). The LI usually resulted in models with the largest differences, and the NA interpolation was the most accurate. Although TT, TD, and TH were not the most accurate, there was another important issue to be considered: the narrower range of data. The most accurate model, based on the median difference, was the one with the NA interpolation with a cloth size of 5 and a threshold of 0.2; however, this model had the highest outliers in a positive direction. The TT, TD, and TH models were the models with minimal differences in medians (and insignificant differences based on the post hoc test).

### 4.4. Effect of the Resolution on the Accuracy

**Figure 9:**
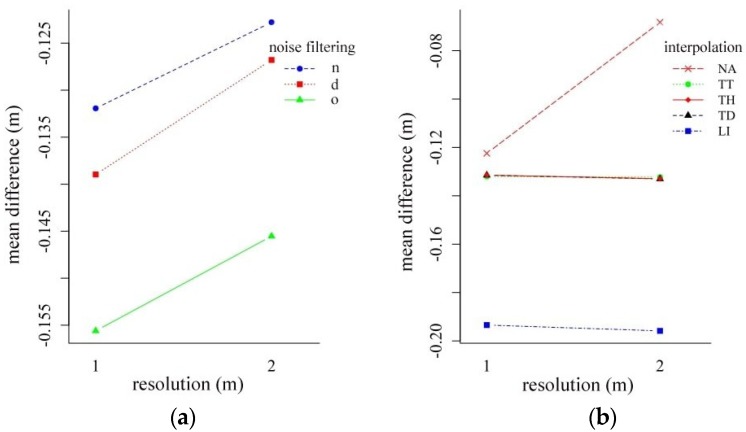
Interaction plot of resolution and noise filtering (**a**) and resolution and interpolation techniques (**b**) (LI: linear interpolation; NA: natural neighbor interpolation; TD: terrain dataset with natural neighbor interpolation; TH: terrain dataset with thinning and natural neighbor interpolation; TT: topo to raster interpolation; d: surface distance-based; n: neighborhood distance-based filtering; o: original point cloud).

We analyzed the dataset from the perspective of the resolution of the final, interpolated maps (Figure 9). The two-way ANOVA revealed that the coarser resolution (2 m compared to 1 m) resulted in more accurate models: models were 0.009–0.012 m closer to the reference surface, on average. Noise reduction and resolution had a significant main effect (Q = 144.8, *p* < 0.001 and Q = 120.1, *p* < 0.001, respectively), but their interaction was not significant (Q = 0.14, p = 929). In the case of interpolation methods, all the main effects were significant (for interpolation Q = 2563.6, *p* < 0.001, for resolution Q = 79.9, *p* < 0.001) and their interaction was also significant (Q = 4478, *p* < 0.001). According to the previous results, LI, TT, TH, and TD interpolations had the same values, and the resolution did not change the differences, but in the case of NA a relevant improvement (0.058 m on average) was observed.

### 4.5. Effects of the Noise Reduction and the Ground Point Classification on the Fluvial Forms

Point bars had larger point/m^2^ values in each model except in two cases (both distance- and neighborhood-based noise reduction models with the cloth size of 5 and the threshold of 0.2). Accordingly, the difference was significant (W = 140; z = 2.373; p_MC_ = 0.015).

**Figure 10:**
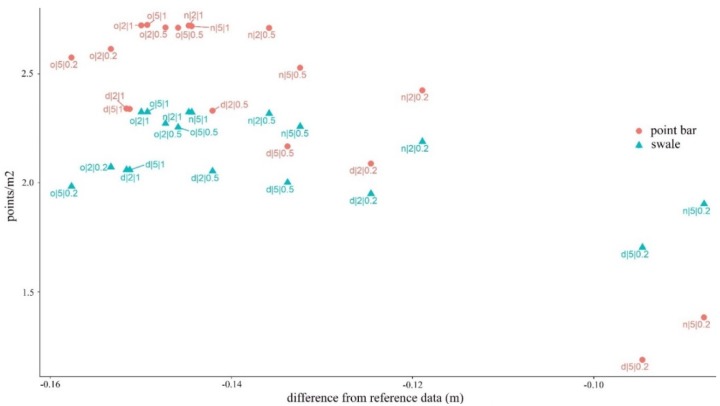
The effect of the noise reduction and the ground point classification on the point bars and swales.

We revealed that the greatest accuracy (i.e., the smaller differences between the reference and measured values) was found in the ground point classifications of smaller points densities: 1.38 points/m^2^ for point bars and 1.90 points/m^2^ for swales (Figure 10). Furthermore, the best CSF parameters were in accordance with the previous results, and the fewest points, the cloth size of 5, and the threshold of 0.2 resulted in the highest accuracy. The noise reduction was efficient, and, although the difference between them was slight (less than 0.01 m), the neighborhood-related filtering was the most effective. Resolution had a significant effect on the accuracy, but the difference was only 0.009–0.011 m between the 1 and 2 m geometric resolution models. Generally, the accuracy of the swales was always below that of the point bars by 0.057–0.069 m.

## 5. Discussion

Aerial LiDAR is a promising technology for collecting large amounts of data even in areas which are hard to access due to dense vegetation or their topography [64]. Surveys result in continuous data from the target areas, and the outcomes are point clouds with several million data points with horizontal and vertical coordinates, the intensity of the returning beams, and, in the case of multiple echoes, the number of returns [65]. Although these datasets represent the fastest and most accurate method of surveying and provide the possibility of object detection beside the elevation data, they have limitations as well [66]. Our study area was a fluvial landscape with swales and point bars, with areas of temporary and locally permanent water cover, and with different vegetation densities (including trees, bushes, grasslands, and aquatic vegetation). These factors bias the final models derived from the point clouds, and we focused on the steps involved in the data preparation and the process of creating a digital terrain model.

In previous works, e.g., [19,28,67,68,69], it was found that noise reduction during preprocessing yielded a better digital terrain model, but on the other hand this procedure could sometimes reduce the accuracy of the model, as [70] found in their study. We had a hypothesis that the noise reduction of the point cloud results in more accurate models in our case, and we pointed out that different noise reduction techniques can have a significant effect on the input data which are the basis of the next stage of the process, i.e., ground point classification. Although the distance-based method including island removal (i.e., deleting points clusters without connection to other points) seemed a powerful method, in the end we found that neighborhood-related noise removal provided the best input data for ground point classification in this fluvial area. However, noise removal was an important step, and both methods resulted in better models than the original point cloud without noise filtering. The difference between the two noise filters is that the distance-based algorithm also removes too many ground points and consequently leaves fewer points for the interpolation phase. Nevertheless, the difference between the noise filtering techniques was small, resulting as 0.01 m on average with same standard deviation (Table 2). Accordingly, we confirmed that noise removal is a relevant beginning step in DTM generation from LiDAR point clouds. Our results are in accordance with the suggestions of [13], showing that in the case of flat terrain, noise filtering based on a statistical approach can be an effective technique. Regarding all combinations, our final DTMs were more accurate using preliminary noise filtering, with slightly better outcomes using the neighborhood distance-based method.

As one of the key steps in the ALS data processing is the point cloud filtering, the number of the different filters for ground point extraction is continuously increasing. In our study we utilized the method of [29]—which applies a cloth simulation filter—where attention needs to be paid to set the most effective parameters, as it requires specific experience. The cloth simulation filter has two important parameters. The first parameter is cloth size, which is in accordance with the point cloud density, i.e., too low and too high values can also result in inappropriate models. We found that models produced from a cloth size of 5 m were more accurate than the finer, 2 m, setting. Thus, larger cloths (larger kernel windows) have more relating points where the algorithm can validate the threshold setting. An important result is that point density was 4 points/m^2^; furthermore, calculating with multiple echoes, it can even reach 10 points/m^2^ [50]. Accordingly, a finer cloth size would have been reasonable, the recommendation of the developer is one-third of the point spacing (http://ramm.bnu.edu.cn/researchers/wumingzhang/english/default_contributions.htm), but according to the mean difference between the two settings, the 5 m cloth was 0.012 m better (for the neighborhood-related filter (Figure 4)). The second CSF parameter is the threshold. A threshold value of 0.5 was suggested by [29], but this was not the best setting in our case. A smaller value of 0.2 resulted in more accurate models with all interpolation methods. We confirmed that, generally speaking, lesser points provided better input for DTM generation, which agrees with the results of [68,69,71]. However, lesser points did not mean the least points. A distance-based filter resulted in the least points, whereas the dataset of the neighborhood-based filter was the best input for the interpolation (Table 2).

In studies by [71,72], the NA algorithm was proposed as the most appropriate one for the representation of coastal subdued areas using a LiDAR point cloud. In our work, interpolations had a relevant effect on the DTMs, and there were significant differences between the methods studied. According to the median-based rank of the differences, the NA had the best and the LI had the worst performance; the TT, TD, and TH models were found in the middle range. The TT, TD, and TH interpolations had very similar results without significant differences (*p* > 0.05). Although NA interpolation (with a cloth size of 5 and a threshold of 0.2 as CSF parameters) provided the lowest differences according to the medians, it had a skewed distribution with a relevant number of outliers in the positive differences. As differences were calculated by subtracting the modeled terrain heights from the reference, this means that the most accurate NA model had several underestimated real heights, while models usually overestimated them. The TT, TD, and TH (without significant differences) showed medians only with slightly lower differences than the best NA, but the ranges were the narrowest. This can be an advantage as the potential error is smaller. However, unlike LI and NA methods, which are embedded in several software packages, including open-source solutions (e.g., ArcGIS [52], GRASS GIS [73], SADA [74], Surfer [75], R [76], and Python [77]) which ensure the widespread usage of the algorithms, TT, TD, and TH interpolations are available only in ArcGIS. Thus, these algorithms can be considered software-specific solutions limited to the users of that software. Differences were not high, but were greater than results of [48], who found differences from the reference data ranging from –0.05 to +0.05 m. In our case, the range varied from –0.10 m to +0.60 m, and we observed large differences in individual points (–1.67 m to +0.62 m, which was relevant in the floodplain, considering that the relative difference between the deepest points of the swales and the highest point of the point bars was only 0.80–1.10 m).

Resolution seemed a significant influencing factor in DTM generation, but without interaction with the noise filtering: the 2 m setting was more accurate by 0.007–0.010 m for each combination of the noise filters or the original dataset. This was in accordance with the analysis of cloth size parameter, where the larger (5 m) size resulted in more accurate models. Furthermore, interpolations were usually insensitive to the resolution, but the NA method, which was considered the best performing technique, was accurate to 0.005 m with the 2 m option. Accordingly, our recommendation is to use the coarser resolution of 2 m. The differences were not high but were significant.

In floodplains, and especially in our study area, the dominant forms were the swales and the point bars. Point bars are always in a higher terrain position in relation to swales; accordingly, swales’ water coverage lasts longer, and the vegetation’s water supply is relevantly better. In our previous study [50], we revealed that the vegetation density is significantly higher in swales; therefore, the number of ground points per m^2^ was significantly smaller (5.88 vs. 4.91). NDVI differed significantly (F = 1567, *p* < 0.001), which was a relevant background factor which influenced the model accuracy when investigating these fluvial forms: removing the dense vegetation from the surface cannot be as accurate for swales as point bars.

Generally, we have to note that these differences were not high, and we can come to the conclusion that, on average, all were in a negligible range. However, as reflected in Figure 7, the spatial appearance of the anomalies can relevantly alter the surface. These small differences can generate a different environment and change the characteristics of the fluvial forms, altering the waterflow modeling. The error propagation is unpredictable; thus, our primary task is to provide the best model and geomorphology seemed a good indicator by which to choose them.

## 6. Conclusions

This study provides a brief description of point cloud processing from noise reduction to digital terrain generation. Our hypothesis was that noise filtering, ground point classification, interpolation, and geometric resolution have significant effects on the generated DTMs. We found that all types of preliminary noise filtering had significantly more accurate results related to the processing than when only applying the original database. CSF as a ground classification technique was a powerful tool, and the resulting DTMs had very low errors (from −0.03 to −0.22 m as upper and lower quartiles). CSF parameters had a significant effect on accuracy, where a coarser cloth size (5 m) and a smaller threshold (0.2) resulted in the best model performance. In the case of interpolations, we have drawn two conclusions: the natural neighbor method provided the most accurate model considering the medians; regarding the range of the differences, the topo to raster and terrain dataset approaches with natural neighbor interpolations provided the best DTMs. Although the density of the ALS point cloud made it possible to use a 1 m geometric resolution for the final DTM, the 2 m resolution was more accurate. We also revealed that landform elements, even when the line of sight is not limited by the topography, can decrease the models’ accuracy; swales had significantly larger model errors due to denser vegetation and water absorption related to point bars. These results proved our hypotheses and can serve as a guidance for ALS LiDAR point cloud preprocessing, classification, and interpolation and for choosing the right resolution in a fluvial environment.

## Figures and Tables

**Table 1:** The parameters of the survey.

Parameters	Value
Designed point density	4 pts/m^2^
Average accuracy (horizontal and vertical)	±0.15 m
Overlap	30–60%
Pulse repetition rate	270 kHz
Registration	discrete return
Laser wavelength	1550 nm
AGL height	688 m
Extent of the surveyed area	126 ha

**Table 2:** Accuracies as reflected in the noise reduction and CSF parameters (o: original LAS dataset; d: distance-based filter with island detection; n: neighborhood-based filter; CS: cloth size; Thd: threshold; SD: standard deviation).

Filtering Method	Noise Reduction	CSF Parameters (CS; Thd)	Point Number	Accuracy (mean ± SD; m)
Original point cloud	-	-	10,120,880	−0.15 ± 0.17
Noise filter	d	-	8,718,994	−0.13 ± 0.15
	n	-	10,073,485	−0.12 ± 0.15
Ground point filter	d	2; 1	6,943,468	−0.15 ± 0.17
	d	2; 0.2	5,199,607	−0.12 ± 0.13
	d	2; 0.5	6,375,149	−0.14 ± 0.14
	d	5; 1	6,875,994	−0.15 ± 0.17
	d	5; 0.2	3,720,552	−0.09 ± 0.16
	d	5; 0.5	5,905,299	−0.13 ± 0.14
	n	2; 1	8,050,253	−0.14 ± 0.16
	n	2; 0.2	5,958,207	−0.12 ± 0.13
	n	2; 0.5	7,395,394	−0.14 ± 0.14
	n	5; 1	7,971,242	−0.14 ± 0.16
	n	5; 0.2	4,246,638	−0.09 ± 0.16
	n	5; 0.5	6,842,756	−0.13 ± 0.14
	o	2; 1	8,293,970	−0.15 ± 0.18
	o	2; 0.2	6,999,426	−0.15 ± 0.16
	o	2; 0.5	7,837,259	−0.15 ± 0.16
	o	5; 1	8,287,750	−0.15 ± 0.18
	o	5; 0.2	6,729,067	−0.16 ± 0.16
	o	5; 0.5	7,781,547	−0.15 ± 0.16

## Supplementary Materials

The datasets analyzed for this study can be found in the Mendeley Data https://data.mendeley.com/datasets/publish-confirmation/d93f3vmdm5/1.
